# The Ubiquitin-Proteasome System: Potential Therapeutic Targets for Alzheimer’s Disease and Spinal Cord Injury

**DOI:** 10.3389/fnmol.2016.00004

**Published:** 2016-01-26

**Authors:** Bing Gong, Miroslav Radulovic, Maria E. Figueiredo-Pereira, Christopher Cardozo

**Affiliations:** ^1^Department of Medicine, Mount Sinai School of MedicineNew York, NY, USA; ^2^Medicine, James J. Peters Veteran Affairs Medical CenterBronx, NY, USA; ^3^National Center of Excellence for the Medical Consequences of Spinal Cord Injury (SCI)Bronx, NY, USA; ^4^Department of Biological Sciences, Hunter College, and the Graduate School and University Center, The City University of New YorkNew York, NY, USA

**Keywords:** ubiquitin-proteasome system, Alzheimer’s disease, spinal cord injuries, beta-amyloid clearance, neuroregeneration

## Abstract

The ubiquitin-proteasome system (UPS) is a crucial protein degradation system in eukaryotes. Herein, we will review advances in the understanding of the role of several proteins of the UPS in Alzheimer’s disease (AD) and functional recovery after spinal cord injury (SCI). The UPS consists of many factors that include E3 ubiquitin ligases, ubiquitin hydrolases, ubiquitin and ubiquitin-like molecules, and the proteasome itself. An extensive body of work links UPS dysfunction with AD pathogenesis and progression. More recently, the UPS has been shown to have vital roles in recovery of function after SCI. The ubiquitin hydrolase (Uch-L1) has been proposed to increase cellular levels of mono-ubiquitin and hence to increase rates of protein turnover by the UPS. A low Uch-L1 level has been linked with Aβ accumulation in AD and reduced neuroregeneration after SCI. One likely mechanism for these beneficial effects of Uch-L1 is reduced turnover of the PKA regulatory subunit and consequently, reduced signaling via CREB. The neuron-specific F-box protein Fbx2 ubiquitinates β-secretase thus targeting it for proteasomal degradation and reducing generation of Aβ. Both Uch-L1 and Fbx2 improve synaptic plasticity and cognitive function in mouse AD models. The role of Fbx2 after SCI has not been examined, but abolishing ß-secretase reduces neuronal recovery after SCI, associated with reduced myelination. UBB+1, which arises through a frame-shift mutation in the ubiquitin gene that adds 19 amino acids to the C-terminus of ubiquitin, inhibits proteasomal function and is associated with increased neurofibrillary tangles in patients with AD, Pick’s disease and Down’s syndrome. These advances in understanding of the roles of the UPS in AD and SCI raise new questions but, also, identify attractive and exciting targets for potential, future therapeutic interventions.

## Introduction

**Figure 1:**
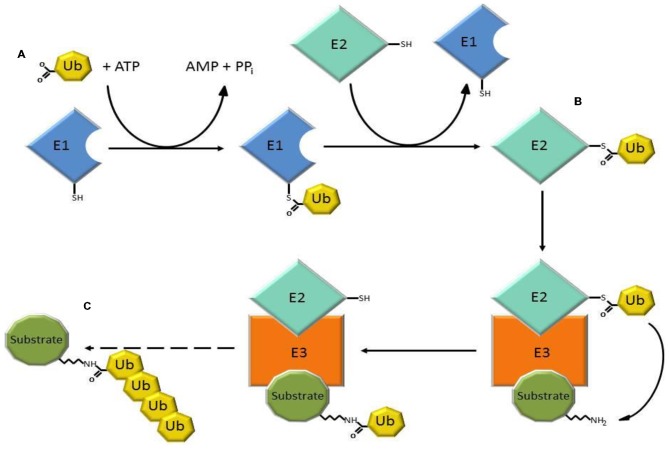
**Three steps of the ubiquitin-proteasome system (UPS) involved in substrate degradation. (A)** Ubiquitin-activating enzymes (E1) form a thioester bond with ubiquitin in a reaction that requires ATP. **(B)** Ubiquitin is transferred to ubiquitin conjugases with which a new, high-energy thioester bond is formed. **(C)** Ubiquitin ligases (E3) provide substrate recognition and catalyze the covalent attachment of ubiquitin to the target substrate via an isopeptide bond. The polyubiquitinated substrate is shuttled to the 26S proteasome for degradation.

The ubiquitin-proteasome system (UPS) is a major intracellular protein degradation system (Schwartz and Ciechanover, 2009). Initially, ubiquitin is activated through the ATP-dependent formation of a high energy thioester bond between the active site cysteine of the ubiquitin activating enzyme (E1), and the carboxyl terminus of ubiquitin. Ubiquitin is then transferred to ubiquitin conjugases (E2), and is finally conjugated to lysine residues within the substrate, or to the N-terminal amino group, by an ubiquitin (E3) ligase, which recognizes specific motifs in the targeted protein. Additional ubiquitin molecules are conjugated onto the first, forming a polyubiquitinated adduct that is recognized by the 26S proteasome leading to substrate degradation (Figure 1; Bence et al., 2001; Adams, 2003).

Dysfunction of the UPS is associated with many neurological diseases including Alzheimer’s disease (AD), Amyotrophic Lateral Sclerosis (ALS), neuronal degeneration, remodeling and regeneration after spinal cord injury (SCI), Parkinson’s disease (PD), Transmissible Spongiform Encephalopathies (TSE), and Huntington’s disease (HD; Leigh et al., 1991; Neumann et al., 2006). The impact of the UPS in these disorders may be related to deficits in the clearance of misfolded proteins leading to intracellular protein aggregation, cytotoxicity, and cell death (Demuro et al., 2005; Schwartz and Ciechanover, 2009). Ubiquitinated proteins are detected in oligomeric beta-amyloid (Aβ) plaques and neurofibrillary tangles, and a mutation in the UPS associated gene UBB+1 causes neuronal degeneration, and has been linked to AD (van Leeuwen et al., 1998; Tan et al., 2007) and to spatial memory impairment (van Tijn et al., 2011).

There are interesting similarities in the role of the UPS in SCI and AD although these neurological conditions differ in etiology, age at onset, and region of the central nervous system (CNS) affected. AD is the most common cause of dementia and is a degenerative disorder of the characterized by memory loss, and neuropathologically by accumulation of Aβ in neurofibrillary tangles. SCI is trauma whereas AD is a degenerative disorder of older individuals. SCI results from traumatic injury to the spinal cord that is most commonly due to motor vehicle accidents, sports or falls, and which results in partial or complete loss of sensation and voluntary movement below the site of the injury. SCI is most common in younger men although a bimodal distribution is now recognized reflecting the growing number of new cases of SCI in the elderly. SCI affects approximately 275,000 individuals in the united states with approximately 12,000 new cases each year. UPS dysfunction is closely associated with the pathological hallmarks of AD including the accumulation of Aβ, formation of Aβ plaques, and hyper-phosphorylation of Tau, which is the major component of intracellular neurofibrillary tangles. The UPS is also involved in neuronal signaling pathways that regulate neurotransmitter release, synaptic membrane receptor turnover and synaptic plasticity (Zhao et al., 2003; Patrick, 2006). Compelling evidence indicates that the UPS also plays a role in the recovery from SCI (Coleman and Perry, 2002). While the accumulation/aggregation of ubiquitinated protein is a biomarker in the early response to SCI due to the impairment of proteasome function (Li and Farooque, 1996; Myeku et al., 2012), an increase in ubiquitin levels is essential for neuronal regeneration after SCI (Schwartz and Ciechanover, 2009; Noor et al., 2011). Furthermore, the UPS also has a role in neuronal development, differentiation, and programming of neuro-stem and neuroprogenitor cells. Regulation of Aβ production by the UPS may have a role in both AD pathogenesis and recovery from SCI. There are several excellent reviews that have discussed the mechanisms and relationships of UPS and tau phosphorylation in AD development (Ciechanover and Kwon, 2015; Gentier and van Leeuwen, 2015; Kumar et al., 2015), and also many reviews that highlight deubiquitination enzymes and the E3 ligases in the role of substrate recognition and ubiquitination. In this review, we have focused on several recent areas of new research: the role of ubiquitin C-terminal hydrolase L1 (Uch-L1), Ubiquitin-B+1 (UBB+1), F-box protein 2 (Fbxo2), and aggregation-promoting chaperones (CRAM-1 and MOAG-4) in Amyloid precursor protein (APP) and Aβ related metabolism as well as the related signaling pathways in AD and SCI, and discuss pressing questions in these fields of research. We also discuss the possibility that one or more of these molecules are potential targets for pharmaceuticals.

## Targeting Uch-L1 and Fbx2 in AD

### Uch-L1 Regulates the CREB Signaling Pathway in AD

UPS dysfunction slows or stops the degradation of many proteins, including misfolded proteins, and results in protein aggregation in the cytoplasm, nucleus and extracellular space of neurons. Formation of inclusion bodies disrupts cell homeostasis, axonal transport, synaptic receptor activity, and neurotransmitter release (Ehlers, 2003; Jarome et al., 2013), and also results in mitochondrial swelling and damage (Maharjan et al., 2014), impaired cell differentiation, degeneration, and neuronal death via apoptosis or necrosis (Schwartz and Ciechanover, 2009; Kaang and Choi, 2012). In neurons, the cyclic adenosine 3′,5′-monophosphate (cAMP)-cAMP-dependent protein kinase (PKA)-cAMP response element-binding protein (CREB) signaling pathway is implicated in synaptic plasticity and cognitive function, and is regulated by the UPS through degradation of the regulatory subunit of PKA (Vitolo et al., 2002). The activation of PKA and the increase of CREB phosphorylation are essential for the formation of stable long term memory (Chain et al., 1999; Fioravante and Byrne, 2011). This signaling pathway has been shown to be compromised by Aβ both *in vitro*, using oligomeric Aβ treated cultured neurons or brain slices, and *in vivo* as evidenced by Aβ overproducing transgenic AD mouse models (Vitolo et al., 2002; Gong et al., 2004; Smith et al., 2009). Consequently, a decrease in PKA-pCREB levels may contribute to impairment of synaptic plasticity and learning function in AD (Gong et al., 2006; Atkin and Paulson, 2014). It was postulated that compounds that enhance levels of pCREB in brain, such as cAMP phosphodiesterases (PDE) 4, 5 inhibitors, have beneficial effects on cognitive function (Gong et al., 2004; Navakkode et al., 2004).

**Figure 2:**
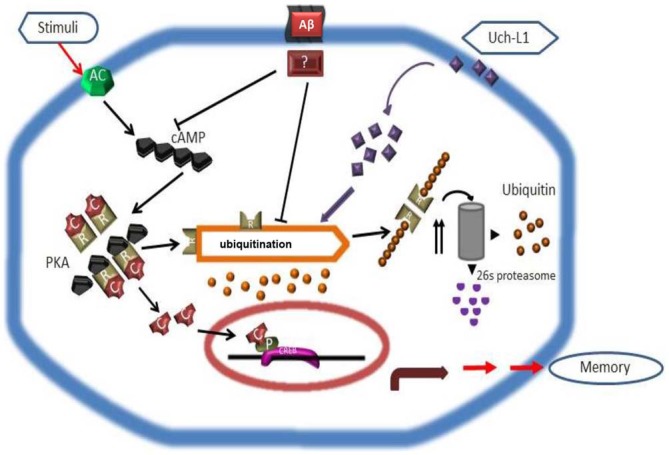
**Scheme depicting how Uch-L1 affects PKA activity, enhances pCREB levels, and improves synaptic plasticity (adapted from Gong et al., 2006).** Aβ inhibits adenylate cyclase (AC) and proteasomal degradation of the RIIa subunit (R), resulting in its accumulation and a shift of the equilibrium in the PKA complex toward the inactive tetramer. As a consequence, the transcription factor CREB cannot be phosphorylated and initiates transcription. Uch-L1 re-establishes normal proteasomal activity leading to normal levels of RIIa subunit which in turn phosphorylates CREB, thus rescuing synaptic function.

In the search for UPS regulators to modulate PKA-pCREB levels in the brain, it was found that in the snail *Aplysia*, the ubiquitin C-terminal hydrolase (Ap-Uch, a homolog of vertebrate Uch-L1) enhances the degradation of the polyubiquitinated R subunit of PKA (Hegde et al., 1997). Uch-L1 is an abundant deubiquitinase (1–5% of soluble protein in brain) that is expressed in neurons, testis and ovaries (Wilkinson et al., 1989; Osaka et al., 2003). Uch-L1 exists as both a soluble form in the cytosol, which accounts for 70–80% of Uch-L1 protein, and a membrane-bound form (Bishop et al., 2014). While the reduction of cytosolic Uch-L1 may be linked to the pathogenesis of AD by contributing to abnormal tau metabolism and Aβ plaque, membrane bound Uch-L1 may be associated with α-synuclein dysfunction in PD (Liu et al., 2009; Chen et al., 2013). Uch-L1 is a highly conserved protein with a catalytic Cys at residue 100 and active site His at residues 107 and 181. The major proposed functions of Uch-L1 are: (1) cleavage of α- and ε-amino acid linked ubiquitin molecules from polyubiquitin chains or polyubiquitinated proteins, respectively (Wilkinson et al., 1989; Larsen et al., 1998) to provide a pool of mono-ubiquitin and regulate ubiquitin homeostasis; and (2) directly binding to mono-ubiquitin to mask mono-ubiquitin activating sites such as Glu64, Phe4, Leu8 and Leu43 (Nakatsu et al., 2000; Shih et al., 2000) to avoid the mono-ubiquitination of substrates that would otherwise shunt them to the endosomal-lysosome pathway to be degraded (Osaka et al., 2003). Uch-L1 C-terminal hydrolase activity can be measured *in vitro* by an enzyme assay using the fluorogenic substrate Ub-AMC, and its activity can be effectively inhibited by the specific inhibitor LDN-57444 (Wilkinson et al., 1989; Gong et al., 2006). Interestingly, Uch-L1 also acts as an ubiquitin ligase and through such activity may have links with PD (Liu et al., 2002, 2006). Studies in AD brains have shown that Uch-L1 deficits are linked to the accumulation of Aβ in the ascending gracile tract as demonstrated by a mouse model of gracile axonal dystrophy (GAD; Osaka et al., 2003), and there is an association between Uch-L1 gene S18Y polymorphisms and sporadic AD (Xue and Jia, 2006; Zetterberg et al., 2010). Uch-L1 levels decrease in postmortem brains of AD patients and in AD transgenic mouse models, coinciding with the accumulation of ubiquitinated protein in Aβ plaques and neurofibrillary tangles (Gong et al., 2006; Zetterberg et al., 2010). Similar to Ap-Uch, human Uch-L1 increases the ubiquitination of the R (regulatory) subunit of PKA in the neuronal cytoplasm by providing mono-ubiquitin to promote proteasomal degradation of the R subunit, thus freeing the PKA catalytic subunit to phosphorylate CREB in the brain (Vitolo et al., 2002; Poon et al., 2013). Uch-L1 also modulates the turnover of the glutamate receptors NMDAR and AMPAR, and has effects on neurotransmitter release (Cartier et al., 2009). Enhancing Uch-L1 expression in the brain could have beneficial effects on synaptic function and improve cognition in AD. This premise is supported by studies showing that overexpressing Uch-L1 or pharmacologically enhancing Uch-L1 activity, improves long term potentiation (LTP) and cognition in AD transgenic mouse models (Gong et al., 2006), whereas knocking out Uch-L1 or pharmacologically inhibiting Uch-L1 causes a decline in cognition (Kurihara et al., 2001; Gong et al., 2006; Chen et al., 2010; Figure 2). This suggests that factors regulating Uch-L1 activity may be potential targets for AD therapeutics. However, more studies are needed to uncover (1) what other signaling pathways that are regulated by Uch-L1 and (2) the effect of such regulatory influences on neuronal function. For example, it remains to be determined how Uch-L1 is involved in the activation of the transcription factor of nuclear factor kappa-light-chain-enhancer of activated B cells (NF-κB) or in tumor necrosis factor (TNF)-induced necroptosis.

### Uch-L1/UBB+ are involved in the Regulation of Aβ Production

Earlier studies failed to demonstrate a significant effect of Uch-L1 manipulations on APP processing and Aβ production in the brain (Gong et al., 2006). However, recent publications show that Uch-L1 is indeed involved in the degradation of APP and beta-site APP cleaving enzyme 1 (BACE1), one of the two essential enzymes cleaving APP and producing Aβ in brain. Overexpressing Uch-L1 reduces BACE1 activity and Aβ levels, whereas Uch-L1 null mice exhibit increased number of Aβ plaques and neurofibrillary tangles, thus displaying an inverse relationship with Uch-L1 levels (Choi et al., 2004; Qing et al., 2004). The discrepancy in the results of various trials regarding the effect of Uch-L1 on Aβ may be due to different dosages and durations of the Uch-L1 treatment in the experimental mice. The mechanism by which Uch-L1 reduces Aβ accumulation and toxicity is unclear. It is postulated that it could be linked to increased APP degradation in Golgi or ER, thus reducing APP levels available to be cleaved by BACE1. Uch-L1 may also affect APP axonal translocation to synaptic terminals, thus reducing Aβ induced neuronal toxicity at the synapse. The latter is consistent with Uch-L1 improving synaptic plasticity in AD mouse models. Thus, there may be multiple pathological mechanisms contribute to the cognitive dysfunction and development of AD. Moreover, drugs may improve cognitive function without altering Aβ levels, which is in agreement with reports suggesting that the clinical phenotype is not always closely related with the Aβ plaque numbers or load in postmortem AD brains (Aizenstein et al., 2008).

A frameshift mutation in the *ubB* (ubiquitin) gene (UBB+1) selectively expressed in brain was associated with increases in neurofibrillary tangles and plaques in AD, Pick’s disease and Down’s syndrome patients, but was not found in the healthy young population, which suggests a link between the UPS and AD pathogenesis (van Leeuwen et al., 1998, 2006). The polyubiquitin chain, which is essential for degradation of most substrates by the 26S proteasome, is formed through isopeptide bonds between the carboxyl terminus of Gly76 of one ubiquitin and the amino group of the lysine residues at positions 48 (K48) of another ubiquitin molecule (Chau et al., 1989). UBB+1 is a result of a dinucleotide deletion during transcription causing frameshift that replaces the glycine at the C-terminus of wild-type ubiquitin with a 19 residue insert (UBB+). The precise mechanism by which UBB+1 influences protein degradation is not clear. While K48 is preserved, UBB+1 cannot form polyubiquitin chains because of the absence of a C-terminal Gly; overexpression of UBB+1 results aggregation of ubiquitinated protein in UBB+1 overexpressing cells (van Tijn et al., 2007) and mice (Irmler et al., 2012). Moreover, UBB+1 conjugated proteins are toxic and directly inhibit 26 proteasome activity, causing aggregation of ubiquitinated protein (Ciechanover and Kwon, 2015). This aberrant UBB+1 has been linked with the accumulation of Aβ and tau in the brain in AD in both post-mortem brains from patients and transgenic mice (Lindsten et al., 2002; Shabek et al., 2009; Dennissen et al., 2010). Studies with AD mouse models showed that knockdown of UBB+1 decreases the steady-state levels of APP, as well as the production of sAPPα, APP C-terminal fragments (CTF), C83 and APP intracellular domain (AICD; Gentier and van Leeuwen, 2015). Conversely, overexpression of UBB+1 increased the levels of APP and Aβ in cultured neuron cells, possibly by reducing the degradation of APP by proteasomes, thus shifting APP into an Aβ production pathway (Massey et al., 2004; Hiltunen et al., 2006; Viswanathan et al., 2011). Therefore, UBB+1 is involved in both secretase-dependent and independent pathways for Aβ generation and this could be a reasonable explanation as to why UBB+ conjugated protein aggregation has been found to be associated with Aβ plaques in transgenic UBB+1 AD brain (van Leeuwen et al., 2006; Zhang et al., 2007; El Ayadi et al., 2012). Since Uch-L1 and UBB+1 exert opposite effects on Aβ processing, metabolism and polyubiquitin chain formation, pharmacological modulation of UBB+1 or Uch-L1 levels in the brain may be a potential therapeutic strategy for AD.

Because Uch-L1 has been found play a role in synaptic function and BACE1 degradation (see below), it will be interesting to know whether UBB+1 has the opposite effect on synaptic plasticity in the AD brain, what signaling pathways are involved, and if UBB+1 also linked BACE1 degradation. Further studies in animal models produced by crossing the UBB+1/Uch-L1 with AD mouse models such as APP or APP/S1 are needed. These studies will provide the basis for screening compounds affecting Uch-L1 and UBB+1 levels that could improve synaptic and cognitive function in AD patients.

### Role of the E3 Ligase Fbox2 in Aβ Processing and Synaptic Function

The UPS is postulated to be involved in the degradation of APP and the attenuation of Aβ accumulation in the brain (Kang et al., 2010; Zhang et al., 2012). Several E3 ligases have been reported to be linked with APP and Aβ processing such as HRDI (Kaneko et al., 2010), the C-terminus of HSP70 interacting protein (CHIP; Kumar et al., 2007) and FBXW7 (Li et al., 2002) which is possibly regulated through the ER associated protein degradation pathway (ERAD). Reductions of E3 ligase function may cause APP accumulation and Aβ generation, whereas, overexpression of these E3 ligases may result in decreased level of Aβ in *in vitro* models. This field has been recently reviewed in depth (Atkin and Paulson, 2014). Here, we will focus our discussion on a newly identified, neuron-specific F-box protein, Fbx2. Fbx2 was found to contribute to the rapid elimination of neuron-specific membrane proteins, including neurotransmitter receptors and postsynaptic scaffolding proteins, by promoting their ubiquitination (Yoshida et al., 2003; Lin and Man, 2013; Atkin et al., 2014). Fbx2 is a member of the SCF-Fbx2-E3 ligase complex. Through its FBA (F-box associated) domain, Fbx2 attaches polyubiquitin chains to high-mannose N-linked glycosylated substrates, thus targeting them for proteasomal degradation via the ERAD pathway (Nelson et al., 2006). Recently, we found that Fbx2 specifically recognizes and binds BACE1 through its FBA domain, thus increasing BACE1 ubiquitination (Gong et al., 2010). Normally, Fbx2 is expressed in various intracellular organelles in brain neurons, and BACE1 is co-localized with Fbx2 mainly in the early endosome and ER (Ko and Puglielli, 2009). Moreover, APP, which is cleaved by BACE as a critical step in the production of Aβ in neurons, is located in the endosome and Golgi complex, and is co-localized with BACE1. During metabolic stresses such as increased oxidative phosphorylation or changes in cytoplasmic homeostasis that induce ER stress, the UPS will be compromised, which would most likely result in a reduction in BACE1 degradation by the UPS and lysosomes. The increased levels of BACE1 therefore are associated with an increase in APP cleavage and overproduction of Aβ (O’Connor et al., 2008; Gong et al., 2010; Singh and Pati, 2015).

We established that Fbx2 overexpression in primary neurons derived from Tg2576 transgenic mice, a well characterized AD mouse model (Hsiao et al., 1995), significantly promoted BACE1 degradation and reduced Aβ production. Not surprisingly, Fbx2 overexpression regulated LTP in the hippocampus. In our *in vivo* studies with Tg2576 mice, we found that stereotactic delivery of adenoviral-Fbx2 to the brain significantly increased Fbx2 expression and decreased synaptic deficits coinciding with a reduction of BACE1, which is essential for Aβ formation (Gong et al., 2010, 2013b). Moreover, we and others demonstrated that in addition to BACE1, the SCF-Fbx2-E3 ligase is involved in binding and ubiquitination of the NMDA and AMPA receptors at the synaptic membrane, affecting receptor turnover during neuronal activation (Kato et al., 2005; Gong et al., 2010). Recently, it was reported that APP is also one of the substrates for Fbx2 ligase (Atkin et al., 2014). APP levels are decreased in the Fbx2 overexpressing cells and increased in hippocampal neurons of Fbx2 knockout mice. The increase in total APP and decrease in surface membrane-bounded APP coincides with higher levels of Aβ in the Fbx2 knockout mice due to subsequent cleavage by β- and γ- secretases.

In summary, there may be two major mechanisms by which Fbx2 regulates synaptic plasticity in AD mouse models: one is through a direct regulation of synaptic neurotransmitter release and receptor turnover; the second may be due to stimulation of the degradation of BACE1/APP to reduce Aβ levels in the brain. Therefore, the Fbx2 E3 ligase could be another potential target for AD treatment. For example, we previously reported that nicotinamide riboside, a potent inducer of Fbx2 expression in neurons, significantly increased the binding of BACE1 to ubiquitin, enhanced BACE1 degradation by proteasome, and thus reduced Aβ toxicity (Gong et al., 2013a). Thus, it would be meaningful to screen compounds that have similar function as nicotinamide that able to boost Fbx2 expression/function in neuronal cells, and then apply these compounds in animal models to test their *in vivo* functions and effects.

### Functional Links between the Ubiquitin-Proteasome System and Mitochondrial Impairment in AD

It is clear that the function of the UPS is perturbed in AD (Ding et al., 2006; Oddo, 2008; Riederer et al., 2011). Defective proteasome activity is detected in the early phase of AD along with synaptic dysfunction, and in late AD stages is associated with the accumulation and aggregation of ubiquitinated (Ub)-proteins which precedes tangle formation (García Gil et al., 2001; Upadhya and Hegde, 2007; Oddo, 2008; Bedford et al., 2009). Mitochondrial impairment is also involved in AD (Eckert et al., 2011; Stranahan and Mattson, 2012; Selfridge et al., 2013). In particular, reduced brain glucose metabolism is detected early in AD patients and is implicated in the initiation and progression of AD pathology (Mosconi et al., 2008). In neurons, even a modest restriction of ATP production by mitochondria seems to far outweigh the effects of reactive oxygen species (ROS; Nicholls, 2008). We will discuss the functional link between UPS and mitochondrial dysfunction, and its impact on AD.

The UPS plays a critical role in the pathogenesis of AD (Riederer et al., 2011). Proteasome impairment could be an upstream event leading to the AD pathogenic cascade. Supporting this notion, we showed that in rat cerebral cortical cultures, short term (4–8 h) proteasome inhibition results in the accumulation of soluble Ub-proteins without significant loss in neuronal viability (Metcalfe et al., 2012). However, longer proteasome inhibition (16–24 h) induces a rise in Ub-protein aggregates, coinciding with caspase-activation, caspase-cleavage of the microtubule associated protein Tau to an aggregation prone form, and neuronal death (Metcalfe et al., 2012). The identity of the factors that induce the transition from soluble to aggregated Ub-proteins is poorly defined. Recent studies with the nematode *C. elegans*, revealed a novel protein known as “cytotoxicity-related aggregation meditaor-1” or CRAM-1 that acts as an aggregation modulator (Ayyadevara et al., 2015). Glutamine/asparagine-rich proteins, which favor hydrophobic interactions with random-coiled domains, seem to be the most susceptible to aggregation (Ayyadevara et al., 2015). CRAM-1 is a highly disordered protein lacking defined protein motifs, and has the ability to sequester unstructured or unfolded proteins. CRAM-1 binds to the polyubiquitin tags of these proteins and blocks their proteasomal degradation and/or targeting to autophagy. This crude sequestration mechanism ultimately leads to an unsustainable aggregate burden (Ayyadevara et al., 2015). Another *C. elegans* protein that plays a similar sequestration role, but functions independent of ubiquitin binding, is MOAG-4 (modifier of aggregation 4) which is homologous to human SERF1 (van Ham et al., 2010). MOAG-4/SERF1 is a specialized and autonomous amyloid factor that promotes aggregation of a broad range of amyloidogenic proteins but is inactive against non-amyloid aggregation (Falsone et al., 2012). Both of these primitive chaperones, CRAM-1 and MOAG/SERF1, impair survival and could act as non-competitive inhibitors of the proteasome in an age-dependent manner relevant to AD (Ayyadevara et al., 2015).

The high levels of oxidized proteins detected in the brains of AD patients are an indirect indication of proteasome impairment, since this proteolytic complex degrades the majority of oxidatively modified proteins (Grune et al., 2004). Impaired clearance of oxidatively modified proteins can cause their aggregation and directly promote the progression of the neurodegenerative process (Grune et al., 2004). Moreover, the accumulation and aggregation of Ub-proteins detected in most neurodegenerative disorders including AD, is also a sign of UPS dysfunction, since this pathway degrades Ub-proteins (Alves-Rodrigues et al., 1998). Impairment in proteolysis mediated by the UPS has profound ramifications for neuronal viability. It leads to the accumulation of modified, potentially toxic proteins in neurons and can cause neuronal injury or premature neuronal death by apoptosis or necrosis (Dasuri et al., 2013). While it is accepted that proteasome function decreases in AD, the question remains why is there a decline in regulated protein degradation by the UPS under these conditions.

One of the emerging factors associated with UPS impairment is mitochondrial dysfunction (Livnat-Levanon and Glickman, 2011). A mechanism by which mitochondrial dysfunction contributes to aging and neurodegeneration is via a net increase in the production of ROS (Zhu et al., 2013). Oxidative stress induced by ROS alters the structure of cellular proteins (Stadtman and Berlett, 1998), which, if not repaired, must be removed by proteolysis to prevent their accumulation and aggregation. Although one of the major roles of the proteasome is to degrade oxidatively modified proteins, whether ubiquitination is required remains elusive (Shang and Taylor, 2011). This is an interesting issue since there is ample evidence that neural tissues are especially vulnerable to oxidative stress, which is linked to AD pathogenesis (Eckert et al., 2011). Neurons exhibit a higher sensitivity to proteasome inhibition than astrocytes, mostly because they exhibit increased levels of oxidized proteins (Dasuri et al., 2010). Interestingly, cysteine deubiquitinases are proposed to be highly sensitive redox biosensors that become inactive and promote a quick response independent of protein synthesis or protein modification (Cotto-Rios et al., 2012; Eletr and Wilkinson, 2014). Furthermore, K63-linked polyubiquitination was proposed to be a new modulator of the oxidative stress response induced by peroxides (Silva et al., 2015). In *S*. *cerevisiae*, three different components of the UPS, the Ub-conjugase Rad6, the Ub-ligase Bre1, and the deubiquitinase Ubp2, were shown to work in concert to regulate K63-linked polyubiquitination of various ribosomal proteins (Silva et al., 2015). Disrupting K63-linked polyubiquitination under peroxide-induced oxidative stress destabilizes polysomes, impairs protein synthesis, and increases the vulnerability of cells to stress (Silva et al., 2015). Thus K63-linked polyubiquitination seems to play a new role as a redox-regulator of protein translation by ribosomes, while K48-linked polyubiquitination regulates protein degradation by proteasomes.

Another deleterious mechanism associated with mitochondrial dysfunction is the limitation in ATP production that can cause an energy crisis in neurons (Nicholls, 2008). Degradation of proteins by the 26S proteasome is highly dependent on ATP binding and hydrolysis (Liu et al., 2006). We investigated the effects of mitochondrial impairment in rat cerebral cortical neurons treated with oligomycin, antimycin, and rotenone, all of which inhibit different elements of the mitochondrial electron transport chain and thus cause a significant decline in ATP (Huang et al., 2013). Our study revealed that a decline in ATP leads to multiple adverse effects: (1) A shutdown of the ubiquitination cascade occurred and was caused by inhibition of ubiquitin adenylation carried-out in an ATP-dependent manner by the E1 ubiquitin activating enzyme; (2) Disassembly of the 26S proteasome was observed and was triggered in part by the selective cleavage of Rpn10 by calpain, which is activated under conditions of ATP deficit. No other proteasome subunit tested was cleaved by calpain. Rpn10 could act as a 26S proteasome gatekeeper sensitive to ATP deficits; (3) There was, accordingly, an increase in the activity of the 20S proteasome, which in concert with immunoproteasomes is postulated to degrade oxidatively modified proteins (Grune et al., 2004); and (4) Cleavage of Tau in a calpain-dependent manner was found, leading to a ~17 kDa fragment which is presumed to be toxic (Garg et al., 2011). These data are highly significant to AD because: (1) mitochondrial dysfunction (Selfridge et al., 2013) and calpain-activation are linked to AD (Saito et al., 1993; Ferreira and Bigio, 2011); and (2) calpain-dependent Tau fragments are detected in the brains of AD patients (Ferreira and Bigio, 2011; Garg et al., 2011). In conclusion, both deleterious consequences of mitochondrial impairment, i.e., restricted ATP generating capacity and ROS production, impair UPS-dependent proteolysis in neurons and are highly relevant to the pathogenesis of AD.

Not only are mitochondria associated with UPS function in regards to ATP production and the generation of ROS, the UPS is also linked to mitochondrial function, as recent studies revealed that the UPS degrades mitochondrial proteins and thus contributes to mitochondrial quality control (Taylor and Rutter, 2011). Mitochondrial proteins that are UPS substrates include: (1) damaged and/or misfolded nuclear-encoded proteins that are destined for import into the mitochondria; (2) defective proteins at the outer mitochondrial membrane (OMM) extracted by p97 and delivered to the proteasome; and (3) non-OMM proteins, although the mechanistic details of how these proteins in the inner compartments retrotranslocate to the OMM remain poorly defined (Farhoud et al., 2012). UPS impairment impacts several aspects of mitochondrial function that include: (1) mitochondrial dynamics (Carlucci et al., 2008) via the scaffold protein AKAP121; (2) mitochondrial biogenesis (Farhoud et al., 2012) via Peroxisome proliferator-activated receptor-gamma coactivator (PGC-1α); and (3) mitochondrial fission via Drp1 (Kanamaru et al., 2012). These factors are turned over by the UPS, and are perturbed in AD by either reduced expression, mis-localization, or an interaction with Tau (Carlucci et al., 2008; Trausch-Azar et al., 2010; Merrill et al., 2011; Manczak and Reddy, 2012; Sheng and Cai, 2012; Sheng et al., 2012).

In conclusion, it is clear that there is a mutual dependance between the UPS and mitochondria (Livnat-Levanon and Glickman, 2011). New discoveries regarding the functional links between UPS and mitochondrial impairment and their roles in overall neuronal homeostasis could lead to novel and successful therapeutic approaches for AD (Huang and Figueiredo-Pereira, 2010). Thus, future studies are needed to better understand how PGC-1α promotes the mitochondrial biogenesis and how the proteasome affects the inner compartments of mitochondrial retrotranslocation to the OMM, and more practically, to test whether stabilization of K63-linked polyubiquitination is able to resist the oxidative stress induced impairment of mitochondrial function in animal models.

## The UPS in Spinal Cord Injury

### Ubiquitin and Uch-L1 Participate in Neuronal Regeneration After SCI

SCI most commonly results from traumatic injuries to the spinal cord. The initial mechanical disruption of white matter tracts is rapidly exacerbated by multiple secondary sequelae that include ischemia, inward calcium flux, infiltration with inflammatory cells and liberation of high levels of ROS which evolve over time to produce a chronic state of inflammation. The acute and chronic production of ROS in the injured spinal cord may be expected to have multiple deleterious effects. ROS are reported to cause oxidation of the catalytic cysteine residue at the active site of E1 and E2 enzymes and promote the formation of E1/E2-containing disulfide complexes, which prevents these enzymes from catalyzing ubiquitination [note: this would decrease levels of ubiquitin conjugates] (Hunter, 2007). Severe oxidative stress also impairs proteasome activity thereby increasing ubiquitin conjugates (Shang and Taylor, 2011). Consistent with the notion that proteasome function is impaired after SCI, ubiquitinated proteins have been demonstrated to accumulate in human spinal cord axons in after SCI (Ahlgren et al., 1996). Ubiquitinated protein aggregates have also been found in neurons of dorsal root ganglia and spinal cord gray matter of rats after spinal cord compression (Li and Farooque, 1996). Selective motor neuron death, induced by transient spinal cord ischemia associated with trauma or surgery, was found to correlate with redistribution of Aβ, ubiquitin, Uch-L1 and the formation of aggregates of proteins in neuronal cytoplasm and nuclei (Yamauchi et al., 2007; Myeku et al., 2012). The increase of ubiquitin and Uch-L1 was observed at the early stage of re-perfusion, after the 15 min spinal cord ischemia, and had resolved by 6 h after re-perfusion in experimental animal models. These findings suggest a strong relationship between vulnerability of motor neurons and the ubiquitin-mediated stress response (Yamauchi et al., 2008).

A critical area of research is understanding mechanisms of neuronal death after SCI and identification of strategies to increase numbers of neurons that survive after the trauma, or so called neuroprotection. Even if damage to the descending and ascending axons in white matter can not be prevented, interneurons present in gray matter may provide a means to rewire spinal cord circuits to restore some connectivity between the cortex and periphery (Courtine et al., 2008). Several lines of evidence link ubiquitin, Uch-L1 and E3 ubiquitin ligases to survival of such interneurons. Specifically, spinal cord interneurons with increased ubiquitin and Uch-L1 expression have been reported to have greater cell survival after SCI (Noor et al., 2013). Similarly, ubiquitin and Uch-L1 up-regulation has also been observed in complete spinal cord transection (Fry et al., 2003; Lane et al., 2007). In addition, vulnerability of motor neurons of the spinal cord can be partially attributed to differences in ubiquitin-mediated stress responses after transient ischemia (Sakurai et al., 1998; Yamauchi et al., 2008). Therefore, a failure to upregulate ubiquitin in motor neurons could represent a great disadvantage for motor neuron recovery (Mengesdorf et al., 2002). Moreover, the ubiquitin E3 ligase ZNRF1 (zinc and ring finger 1) has been demonstrated to be associated with Wallerian degeneration after SCI (Araki and Milbrandt, 2003). Thus the significant changes in ubiquitin expression and cellular distribution in response to SCI, and its effects on the neuronal regeneration and myelination, suggest that the UPS could have a significant role in recovery from SCI caused by ischemia.

Once the acute inflammatory response to SCI has resolved, spinal cord neurons engage in a process of rewiring (neuroplasticity) and in efforts to extend new axons across the injured region (neuroregeneration). Studies in Monodelphis domestica and mice suggest a linkage between neuroregeneration and expression levels of components of the UPS, although the underlying mechanisms are poorly understood. For example, following spinal cord transection in Monodelphis domestica, axonal regeneration and regrowth of neuronal tissue was found at the injury site in animals injured at 7 days after birth (P7), which was associated with upregulation of ubiquitin mRNA and protein expression. However if injury occurred at postnatal 28 day (p28), little or no upregulation in ubiquitin expression occurred and no neuronal regeneration was observed (Ji et al., 2008; Yan et al., 2010; Noor et al., 2013). The levels of mono-ubiquitin were decreased at sites both caudal and rostral to the SCI injury site in aged animals, which also have reduced neuroregenerative capabilities; the magnitude of the decrease was found to be age-related. The reduction in ubiquitin levels may also be associated with involvement of ubiquitin in multiple functions and substrates in an organism outside of the proteasome system such as receptor internalization and modifications to chromatin structure (Noor et al., 2011). Moreover, the distribution of ubiquitin in response to the SCI also plays a role in the recovery of the spinal cord. For example, in motor neurons or interneurons of the ventral horn, ubiquitin is significantly increased in the cytosol after injury of postnatal 7 day (p7) mice which demonstrate significant neuroregenerative capabilities. By contrast, in mature mice, where the capacity of neuron regeneration is lost, there is minimal change in ubiquitin levels in the cytosol of ventral horn neurons, in which ubiquitin was primarily localized to the nuclei (Noor et al., 2011, 2013). These findings suggest that the growth inhibitory or stimulatory factors that explain the difference in neuroregenerative capacity between young and old mice regulate ubiquitin expression and localization and support the view that certain levels of ubiquitin in injured neurons are essential for neuron regeneration. An open question is whether certain deubiquitinating enzymes such as Uch-L1 could be beneficial for the SCI. It is notable therefore that Uch-L1 has been reported to promote neurogenesis and regulate differentiation of neuronal progenitors and has been proposed as a potential stem-cell based therapeutic target in the SCI (Sakurai et al., 2006; Naujokat, 2009).

Several neuroprotective drugs have been shown to reduce accumulation of aggregated, ubiquitinated proteins. Elevation of cAMP in rat spinal cord neurons by rolipram, a PDE4 inhibitor, or dibutyryl-cAMP, is neuroprotective and is associated with the increase in proteasome function by enhancing 19S regulatory particle ATPase 6 (Rpt6), and β5 subunit of the central beta-rings of 20S proteasome activity and other components of the UPS including UBB and the E3 ligase CHIP, resulting in a decrease in aggregation of ubiquitinated protein in SCI (Myeku et al., 2012). Since Uch-L1 enhances the down-stream signaling pathways of cAMP by acting on PKA, this provides another rationale for the targeting of Uch-L1 in SCI treatment.

In summary, the UPS is impaired after SCI, and ubiquitin levels are positively associated with neuroregeneration. While it is logical to assume that elevation of Uch-L1 levels is beneficial after SCI, this possibility has not been tested. Further animal studies are needed in SCI models to determine if the overexpression of ubiquitin or Uch-L1 can lead to resistance to neural injury or promote neuroregeneration or neuroplasticity with benefits to functional recovery.

### The Role of Proteasome After SCI

Dysfunction of the proteasome may explain or contribute to the accumulation of ubiquitinated proteins after SCI (Myeku et al., 2012). However, the role of the proteasome in neuronal regeneration is controversial and likely complex. It has been found that treatment with proteasome inhibitors such as MG132 or lactacystin induces secondary axotomy of stretch-injured axons 72 h after the injury (Du et al., 2008), and arrests neurite outgrowth in primary cultured neurons (Laser et al., 2003). The axonal stretch model replicates the mechanical forces acting on brain or spinal cord following traumatic brain injury (TBI) or SCI (Staal et al., 2007). Of relevance to traumatically induced SCI, motor neurons are also more vulnerable to proteasome inhibitor induced neurotoxicity, which includes apoptotic nuclear changes and mitochondrial dysfunction (Kikuchi et al., 2002). In a rat SCI model, cAMP induced PKA activation reduced the accumulation of ubiquitinated protein that was caused by proteasome inhibition in the spinal cord, thus demonstrating a potential therapeutic approach in the prevention of neuronal damage after SCI (Moore and Kennedy, 2006; Myeku et al., 2012), and raising the possibility that Uch-L1 and PDE4 inhibitors may be beneficial after an SCI.

On the other hand, the drug bortezomib, a 26S proteasome inhibitor currently used in treatment of multiple myeloma, was found to be effective in reducing brain ischemia size in clinical studies (Shah and Di Napoli, 2007) by increasing ubiquitin expression in the infarct. One might posit that treatment of bortezomib could also be beneficial after SCI to preserve motor neurons and to improve neuronal function (Henninger et al., 2006). Furthermore, following nerve transection, MG132, a peptide aldehyde inhibitor of the proteasome as well as multiple cysteine and serine proteases, delays the onset of Wallerian degeneration (Zhai et al., 2003). Similar to the immunohistological studies, microarray analysis showed that inhibition of the proteasome may have both neuroprotective and pro-apoptotic response in primary neuron cells through upregulation of neuroprotective heat-shock proteins (HSPs; Yew et al., 2005), of which the main subtype expressed in motor neurons of spinal cord are Hsc70 and Hsp27 (Chen and Brown, 2007). Of interest, the induction of Hsc70 by proteasome inhibitors prevents neuregulin-induced motor nerve demyelination and improves nerve conductance; these changes may be related to the clearance of c-Jun (Li et al., 2012). However, proteasome inhibition activates antioxidant and HSP signaling pathways, which may result in neuroprotection early after SCI and an apoptotic response at later time points (Yew et al., 2005; Yang et al., 2006). The effects of proteasome inhibitors are thus complex, and likely depend on specificity of the inhibitor used and cell type, but also may be influenced by duration and timing relative to the initial spinal cord trauma. Additionally, proteasomes participate in myriad cellular processes in addition to housekeeping functions such as disposal of damaged proteins. Differences in results of studies with proteasome inhibitors may reflect the relative activation state of one or more signaling pathways regulated by proteasomes, or of expression levels of E3 ligases or other components of ubiquitin machinery. Such differences could explain, in part the divergent and at times apparently contradictory findings with proteasome inhibitors. Thus, further studies in animal models of SCI are needed to explore the role of proteasomes in neuroprotection and neuroregeneration and to define the timing of administration of proteasome inhibitors in the protection of spinal neuron recovery.

### The Role of Ubiquitin Ligases, APP and Aβ in Recovery After SCI

As noted above, the UPS plays critical roles in modulating levels of APP and Aβ. Understanding of the role of Fbox2 or other E3 ubiquitin ligases in neuroregeneration or injury to spinal cord neurons after SCI is limited to extrapolations from our understanding of the role of these factors in Aβ metabolism and AD pathogenesis outlined above. There is some understanding of relationships between APP, Aβ and outcomes after SCI which allows greater understanding of how E3 ubiquitin ligases might be expected to influence neuroprotection or neuroregeneration after SCI. APP is carried along the axon by fast axonal transport. It exists in both pre- and post-synaptic sites and has a role in synaptic function and axonal protein transport. Generation, processing and resultant levels of APP have been identified to be very sensitive to CNS insults and have been extensively used as a biomarker of traumatic axonal injury. Levels of APP immunostaining provide a gold-standard for clinical identification of diffuse axonal injury both in brain and SCI (Blumbergs et al., 1995; Nashmi and Fehlings, 2001). Experimental sciatic nerve axotomy showed an axonal accumulation of Aβ and promotion of the pathological formation of Aβ plaques in the motor neurons in gray matter and the glial cells present in white and gray matter of the spinal cord, which may be a reactive response (Ahlgren et al., 1996; Rose and Odlozinski, 1998). However, inhibition of Aβ accumulation by inhibiting APP-γ-secretase and BACE1 was found to reduce the recovery after SCI indicating that Aβ may also have neuroprotective role after SCI (Ahlgren et al., 1996; Rose and Odlozinski, 1998; Pajoohesh-Ganji et al., 2014). At 1 day after SCI, accumulation of APP and Aβ, the loss of the cytoskeletal proteins such as microtubule associated protein-2 (MAP-2), and axonal demyelination have been found both within and away from injury epicenter (Li et al., 1995; Huang et al., 2007). Such findings suggest that both the initial injury and subsequent secondary injury caused by the activation of cascades which spread the injury away from the trauma site are involved in the above pathological changes. The accumulation of APP in traumatized axons at the epicenter of the injury, and also rostral and caudal to it, correlates with loss of motor neuron function, further demonstrating the crucial link between APP and the SCI. These findings implicate impairment of both fast anterograde and retrograde axonal transport (Medana and Esiri, 2003; Ward et al., 2014), which causes an accumulation of APP, other axonal transport proteins, intracellular components such as lysosomes, neurotrophin, mitochondria and synaptophysin-carrying vesicles. Transport of these components to synapses is essential for normal neuronal function and survival (Lazarov et al., 2007) and could be Aβ independent (Stokin et al., 2008). On the other hand, the build-up of APP in axons and at synapses could cause rapid local increases in Aβ levels following the increased cleavage of APP by γ- and β- secretase. The increased Aβ further impairs axonal transport causing axonal swelling due to damage to axonal microtubules. Similar to the reported toxicity of Aβ in brain, accumulation of Aβ in spinal cord neurons disrupts intracellular calcium homeostasis, impairs mitochondrial function, and results in abnormal regulation of synaptic receptors and ion channels (Pike et al., 1993). It also induces the release of cytokines causing neuronal inflammation and defective endo-lysosomal trafficking (Pimplikar et al., 2010), altered intracellular signaling cascades, or impaired neurotransmitter release thereby contributing to synaptic dysfunction (Tang-Schomer et al., 2010). Thus, drugs regulating ubiquitin expression after spinal injury could have the potential to spare neurons from injury by minimizing Aβ accumulation (Yokobori et al., 2015).

In summary, Aβ accumulation is an early response after SCI, and its accumulation affects neuronal recovery via similar mechanisms to those involved in AD, such as impairing axonal transport, activating cytokine induced inflammation and impairing neurotransmitter release in spinal cord neurons. Further studies are needed to define which signaling pathway is involved in Aβ induced neuronal dysfunction, if the sensory neurons and motor neurons are shared same mechanisms, and the role of the UPS in the regulation of Aβ accumulation in SCI.

### Role of BACE1 After SCI

Increased accumulation of APP and Aβ have been reported in axons in white matter about 1 mm from the lesion site after SCI in mice (Kobayashi et al., 2010). There is evidence that in APP overexpressing mice (TgCRND8 mice), there were increased levels of Aβ at the synaptic terminal and extracellular accumulation of Aβ, which were predominately located in the gray matter of the dorsal horn and the central part of dorsal column of the spinal cord. The enhanced Aβ accumulation coincides with loss motor neurons (Xie et al., 2000). In ALS patients, it has been found that Aβ co-localized with oxidative damage markers such as heme oxygenase-1, and nitro-tyrosine in abnormal neurons and an increased cleaved caspase-3 immunoreactivity in motor neurons (Calingasan et al., 2005).

**Figure 3:**
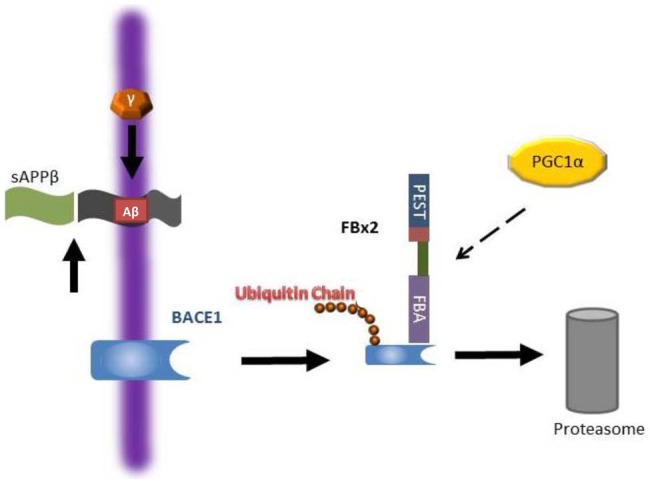
**Working hypothesis as to how Fbx2 facilitates beta-site amyloid precursor protein cleaving enzyme 1 (BACE1) degradation.** In the Alzheimer’s disease (AD) brain, BACE1 is essential to cleave amyloid precursor protein (APP) to generate Aβ. Fbx2 binds BACE1 through an F-box domain and promotes BACE1 ubiquitination and proteasome degradation. PGC-1a stimulates BACE1 metabolism through the enhancement of Fbx2 gene/protein expression and reduces Aβ production. AD, Alzheimer’s disease; APP, amyloid precursor protein (Adapted from Gong et al., 2010; Aging Cell).

BACE1 is an aspartyl protease of the pepsin family that is mainly expressed in neuronal late Golgi and trans-Golgi (TGN) compartments as well as the cytoplasmic membrane (Vassar et al., 1999; Yan et al., 2001). Since BACE1 is an essential enzyme for Aβ generation as described above, in the past decade, many compounds have been developed to block BACE1 activity. Immunostaining showed that BACE1, APP, and Aβ are closely co-localized with large motor neurons in the ventral horn of the spinal cord in AD mouse models and that Aβ plaques are largely derived from the dystrophic presynaptic axonal terminals originating from glutamatergic neurons arising in the brain and projecting to spinal cord neurons (Li et al., 2013). Moderate inhibition of BACE1 activity reduces Aβ production, enhances clearance of myelin debris and improves axonal regeneration after SCI (Farah et al., 2011). However, completely abolishing BACE1 by knocking out the BACE1 gene has adverse effects on recovery after SCI such as delayed myelination and decreased myelin thickness (hypomyelination) due to a dysregulation of neuregulin-1 type III, an EGF-like growth factor (Hu et al., 2006; Willem et al., 2006). Moreover, animal studies have shown that complete inhibition of BACE1 activity results in an elimination of Aβ in neurons, but Aβ is required for certain physiological neuronal functions such as regulation of neuron-glia signaling by enhancing spontaneous astrocyte calcium transient signaling via α7-nAChRs (Lee et al., 2014), activation of M2 microglia cells after SCI promoting plasticity, axonal outgrowth and inhibition of inflammation (Kigerl et al., 2009; Pajoohesh-Ganji and Byrnes, 2011). Since both accumulation and complete elimination of BACE1/Aβ adversely affect recovery after SCI, biological regulation of Aβ or BACE1 levels after SCI by modulation of components of the UPS such as Fbx2-Uch-L1 via peroxisome proliferator-activated receptor-gamma co-activator (PGC)-1alpha or gene expression (Gong et al., 2010, 2013b) may be a feasible strategy for the SCI treatment and AD (Figure 3).

In summary, BACE1 plays multiple, critical functions after SCI. Reducing BACE1 expression prevents the accumulation of Aβ in neurons after SCI, while completely blocking BACE1 activity may result demyelination of axons in white matter tracts. Thus future *in vivo* studies are needed to test when biologically expression of ubiquitin/Uch-L1 as well as other components of the UPS could beneficial to the recovery of SCI.

## Summary

Multiple lines of evidence indicate that dysregulation of any of several components of the UPS, or mutations in genes that encode them, exacerbate AD progression and are deleterious to the recovery of function after SCI. It is notable that many UPS components such as ubiquitin, Uch-L1, Fbox2 and the proteasome itself are critical determinants of disease severity in both SCI and AD. There are, however, many questions to be answered regarding the mechanisms by which these UPS components contribute to AD progression and facilitate or impede recovery of function after SCI. Finally, ubiquitin, Uch-L1, and the proteasome can be manipulated pharmacologically providing convenient methods for research applications and suggesting the possibility that further efforts in this field should include investigation of the potential utility of drugs that target these molecules. The major obstacle in developing such therapies will be the ubiquitous presence of the UPS in eukaryotes which may require nanomedicine approaches or other tissue-targeting strategies to minimize adverse effects of such therapies.

## Author Contributions

BG, MR, MEF-P and CC wrote this article. BG and CC did the revision.

## Conflict of Interest Statement

The authors declare that the research was conducted in the absence of any commercial or financial relationships that could be construed as a potential conflict of interest.
